# A call to create evidence-based mental health promotion interventions for youth that are equitable across ethnic/racial subgroups: Advocates 4-ALL Youth

**DOI:** 10.3389/fpubh.2023.1139921

**Published:** 2023-04-21

**Authors:** Jill L. Kaar, Anne E. Bowen, Stacey L. Simon, Adefunke Dadematthews, Jessica L. Chandrasekhar, Rashelle Musci, Melissa Pangelinan

**Affiliations:** ^1^Department of Pediatrics, University of Colorado School of Medicine, Aurora, CO, United States; ^2^Children’s Hospital Colorado, Aurora, CO, United States; ^3^School of Kinesiology, Auburn University, Auburn, AL, United States; ^4^Department of Mental Health, Bloomberg School of Public Health, Johns Hopkins University, Baltimore, MD, United States

**Keywords:** youth, mental health, school-based action research, equitability, prevention

## Abstract

**Background:**

Adolescents from historically racial and ethnic minoritized and low-income communities have higher rates of early-life and chronic difficulties with anxiety and depression compared to non-Hispanic White youth. With mental health distress exacerbated during and in the wake of the COVID-19 pandemic, there is a need for accessible, equitable evidence-based programs that promote psychological well-being, strengthen one’s ability to adapt to adversity, and build self-efficacy prior to adolescence.

**Methods:**

An evidenced-based resiliency-focused health coaching intervention was adapted using a health equity implementation framework to meet the needs of a Title I elementary school in rural Alabama (AL) that serves over 80% Black and Hispanic students. To ensure that the program met local community needs while maintaining core program educational activities, all adaptations were documented utilizing a standard coding system.

**Results:**

Leveraging an existing academic-community partnership with Auburn University and a local AL school district, a new program, Advocates 4-All Youth (ALLY), was created. Three major adaptations were required: (1) the use of local community volunteers (ALLYs) to deliver the program versus health coaches, (2) the modification of program materials to meet the challenge of varying levels of general and health-related literacy, and (3) the integration of the Empower Action Model to target protective factors in a culturally-tailored delivery to ensure key program outcomes are found equitable for all students.

**Conclusion:**

With continued increases in youth mental health distress, there is a need for the development of universal primary prevention interventions to promote mental well-being and to strengthen protective factors among youth from historically disadvantaged backgrounds. ALLY was created to meet these needs and may be an effective strategy if deemed efficacious in improving program outcomes.

## Introduction

Inequality in mental health care access among racially and ethnically minoritized (1, 2) (e.g., Black and Hispanic/Latino) and low-income (e.g., access to free/reduced cost school lunch) youth is well-documented (3–11). Racially and ethnically minoritized low-income youth face multiple challenges from discrimination to unequal access to relevant and effective mental health services, including programs that promote positive mental health-related outcomes. School-based delivery has been identified *via* research as the most critical location for mental health prevention programs (12). However, such programs have been primarily tested in schools with predominately non-Hispanic White students (13). Thus, accessible, evidenced-based interventions that are effective for preventing depression symptoms, anxiety symptoms, and stress by improving protective factors specifically among racially and ethnically minoritized low-income youth are needed.

Universal prevention efforts delivered within schools have the potential for widespread reach, including students from predominantly racially and ethnically minoritized low-income communities and those who may be classified as “just under the clinical radar” for diagnostic criteria. As students spend most of their waking hours in school, schools can alter the environment of many youths simultaneously and, when implemented successfully into the school’s structure, programs have the potential to be pragmatically integrated and sustained long-term. Moreover, when programs are offered and available to all youth, school-based mental health interventions are typically experienced as non-stigmatizing and demonstrate strong buy-in from schools, parents, and youth stakeholders (13).

Existing school-based programs aimed at reducing stress, symptoms of depression, and anxiety have been effective (13). However, the sustainability, accessibility, and scale up of such programs have been major limitations for widespread implementation within school systems. To date, programs have focused on targeting youth with elevated levels of depression/anxiety which requires screening all students. This model can be expensive, stigmatizing, and difficult for schools to sustain long-term (13, 14). Schools with limited mental health staff and resources are not able to utilize such programs as they do not have the capacity to help the students who report some signs or symptoms but do not meet the program’s specific clinical inclusion criteria. Further, due to the nature of screening, targeted programs are viewed as stigmatizing to students and, therefore, less appealing to school administrations. Lastly, current programs may amplify existing mental health disparities within racially and ethnically minoritized communities as schools in lower income or rural areas may not have the access or the funding necessary to implement such programs.

The objective of this study is to bring attention to the inequality in mental health care and mental health disparities among racially and ethnically minoritized youth, document the systematic process of adapting an evidenced-based resiliency-focused health coaching intervention from a suburban, middle-class, predominately non-Hispanic White school to a new setting with socioeconomical and cultural differences (i.e., rural, low-income, predominately non-Hispanic Black), and to discuss our lessons learned and a path forward for other researchers to ensure programs are developed with intentionality to provide equity of program implementation and access.

## Methods

### Conceptual framework

#### Program framework for intervention

Advocates 4-ALL Youth (ALLY) has been created using best practices and lessons learned from the pilot program, Building Resilience for Healthy Kids (Healthy Kids) (15–17). Healthy Kids was created and piloted in a local Colorado community with key school and community stakeholders and designed for widespread dissemination using a pragmatic approach that can be highly personalized within the existing school structure. Healthy Kids has been evaluated by key stakeholders *via* both qualitative and qualitative methods and was found to be highly acceptable to students, teachers, principals, and administers. The program offered delivery flexibility in terms of the weekly, 30-min sessions to accommodate student/school schedules while minimizing missed sessions. As a universal program, Healthy Kids was designed with the intention that all students participate in the program and, to ensure buy-in from youth, parents, and the school community, the sessions have been incorporated into the existing school curriculum. Pilot data demonstrated improvements in symptoms of depression and anxiety among students who reported elevated symptoms at baseline, while improving resilience and self-efficacy among all students (16).

ALLY was created to extend the positive pilot findings of Healthy Kids by adapting program materials and delivery to meet needs of a new community of students. Specifically, ALLY has focused on utilizing tailored programming of materials to ensure program outcomes previously documented in one community were equitable among students in this new community which differed according to racial, ethnic, and cultural differences. Further, we focused on sustainability of the program within this new existing school systems with little to no financial or staff burden. ALLY utilizes community volunteers (ALLYs) with no previous health-related training required, compared to the certified health coaches used in Healthy Kids. The use of ALLYs to deliver evidenced-based mental health programs rather than mental health para-professionals has been suggested as a key approach to address mental health disparities for underserved populations (18). This approach has been shown to be more acceptable and feasible for schools with limited access to mental health resources (19–21).

The development of ALLY was guided by two well-known theoretical frameworks, the Socio-Ecological Model and the Social-Ecological Suicide Prevention Model. The Socio-Ecological Model (22) is shown to provide a sound guiding theoretical framework for improving social connectedness *via* community and school support. Improving social connectedness is particularly vital for racially and ethnically minoritized and low-income youth as recent data (23) found that the mental health of these students were most affected by the lack of social connectedness due to school closures and social isolation (24, 25).

The adapted Social-Ecological Suicide Prevention Model posits that suicide prevention programs need to incorporate targets for improvement of protective factors (i.e., self-efficacy, resilience) at the individual level. Increasing youth’s protective factors such as self-efficacy and resilience have been found to assist with prevention of worsening anxiety and depression (26, 27). Symptoms of anxiety and depression among youth have been consistently negatively related to self-efficacy. Resilience, i.e., the process of adapting in the face of adversity and the ability to bounce back from stressful experiences (28–30), is an individual protective factor against negative affectivity and suicidal ideation, but it is influenced by youths’ perception of social support from adults in their community (31).

When an individual’s school environment is focused on the interrelationship between the social setting and a youth’s well-being, youth tend to develop a strong foundation for positive development through adolescence and early adulthood (32). Early adolescence is a developmental period that is sensitive for altering the course of self-efficacy and resilience and, in turn, deterring increased negative affectivity and suicidal ideation. Thus, interventions timed in early adolescence, or the start of middle school, offer the potential to reduce future suicide risk by bolstering self-efficacy and resilience and preventing the worsening of negative affectivity. ALLY is universally delivered, school-based program designed to reach racially and ethnically minoritized low-income youth with a goal to eliminate inequality in mental health illnesses and mental health care access and, ultimately, suicide.

#### Study overview

The program, Building Resilience for Healthy Kids (Healthy Kids), was developed using a community-based participatory research approach and served as a proof of concept and pilot-tested in Colorado (15–17). Healthy Kids was a single-arm intervention delivered in January–March 2020 to 6th-grade students attending an urban public middle school. The pilot sample was 54% female, 16% qualified for free or reduced cost lunch (a proxy for socioeconomic status), and 28% were classed as historically racially and ethnically minoritized (i.e., African American, Hispanic) youth. In July 2021, Healthy Kids transitioned into ALLY as it moved to a new cultural setting located in a community in rural Alabama. The new school population was 47% female, 88% free-reduced lunch, and 88% racially/ethnically minoritized youth. All study procedures involving participants were approved and implemented in accordance with the ethical standards of the Colorado Multiple Institutional Review Board (COMIRB # 21–3,720 and clinical trials #NCT05025657).

#### Conceptual framework for adaptations

Here, a health equity implementation framework was used to document program adaptations in a new setting (33). Specifically, we used the FRAME framework for reporting adaptions and modifications along with the coding system explained by Rabin et al. (34, 35) ALLY has been designed for dissemination. As the program is pilot-tested within new communities across the U.S., such modifications will be documented to ensure program materials are adapted to local cultural styles, appropriate for average literacy level, and delivered in a format that is sustainable within the schools’ existing system characteristics (36).

#### Adaptation framework

To ensure our program met local community needs while maintaining core program educational activities, we documented all adaptations using the Rabin et al. adaptation framework and coding system (34). This process aims to effectively adapt interventions to new settings with demographic differences. The Stirman et al. system of categorization of adaptions was also utilized to document type of modification (i.e., context or content) (35). Contextual modifications were then categorized as format, setting, personnel, or population. Content modifications included categories such as tailoring/tweaking/refining, changes in packaging or materials, adding elements, and lengthening/extending (pacing/timing). A full description of this framework has been published by Stirman et al. (35).

#### Data collection

Multilevel stakeholder engagement (school administration, teachers, program facilitators (i.e., interventionists), students, and researchers) provided feedback throughout the process. Feedback was documented at the level of each adaptation including a reason for the modification, identified person(s) that provided feedback, description of the modification, level of delivery of modification, content or context of modification, and the impact of the modification.

## Results

An overview of the program modifications that occurred in the transition from Healthy Kids to ALLY are summarized in Table 1. Both contextual and content modifications occurred and are summarized in Tables 2, 3. All modifications focused on the goal to improve effectiveness, adoption, and engagement of ALLY by the school and local stakeholders.

**Table 1:** Summary of program modifications from healthy kids to ALLY.

Type	Healthy kids	ALLY
Contextual modifications		
Setting: School	Middle (6th grade)	Elementary/Middle (5-6^th^ grade)
Population: Free and reduced lunch	16%	88%
Population: Average minority student enrollment	28%	88%
Personnel: Facilitators	Certified health coaches	Community volunteers
Format: Materials	Open format – No set material	Workbook
Content modifications		
Tailoring	6th grade reading level	3^rd^ grade reading level
Lengthening/extending	6 weeks	6–10 weeks (Up to 15 weeks)
Integrated framework	Cultural humility	Empowered Action Model

**Table 2:** Contextual modifications made to improve effectiveness, access/reach, adoption, and engagement of program by school.

Modification	School setting	Literacy	Community volunteers	Workbook
Description	6^th^ graders remain in elementary school in this community & attend classes with 5^th^ graders	Held seminar for teachers to review intervention materials and ask questions regarding differences in language used (i.e., defining bullying)	Local community members were trained as facilitators (i.e., “ALLYs”) to ensure that the program could be delivered and sustained at the community level	Students work through specific assignments during the intervention period
Reason	Ease of scheduling students	Program sustainability	Program sustainability	Consistent intervention delivery
By WHOM	Partnership with school and researchers	Partnership with school and researchers	Partnership with local University, school, and researchers	ALLYs, Researchers
WHAT	Content of program materials	Content of program materials	ALLYs’ training	Content of program materials
Level of DELIVERY	Student	Student	ALLYs	Student
CONTEXT	Setting	Population	Personnel	Format
Potential IMPACT	5–6^th^ grade students were included due to scheduling needs	Teachers and school staff were engaged and openly communicated language differences and aligned with program	8 ALLYs trained	ALLYs reported the workbook kept the practice sessions more focused and felt more able to teach the materials to students. ALLYs also reported more confidence in delivering the program compared to using the CO-based program materials

**Table 3:** Content modifications made to improve effectiveness, access/reach, adoption, and engagement of program by school.

Modification	Reading level	Six sessions	Equity framework
Description	Reading level modified from 6th grade to 3rd grade	Students must complete all six sessions in any amount of time during the semester	Incorporated cultural humility focus throughout ALLYs’ training
Reason	Average reading level of student population was 3rd grade	We know this is an effective number of sessions from our pilot; feasibility issues with school to fit in all six sessions due to student absences	To address mismatch between facilitator background and identities and that of the student population/school setting
By WHOM	Teachers, school counselors	Researchers in partnership with school feedback	Researcher, collaborators at Auburn University
WHAT	Student materials	Length of intervention period	ALLYs’ training materials
Level of DELIVERY	Student	Student	Researchers trained
CONTENT	Tailoring materials: reading level	Extending timing	Integrated a new framework: Empowered Action Model
Potential IMPACT	Improved engagement of students	More time for all students to complete the full program	ALLYs feeling more connected to their students and vice versa

### Contextual modifications

The 6^th^ grade students in this community remained in elementary school and attended classes with the 5^th^ grade students. In partnership with the school and researchers, ALLY was modified at the level of the student to ensure that the content of program materials was age- and reading-level appropriate. The primary reason for this change was to ensure that the school could accommodate the individual sessions within their existing schedule for both 5^th^ and 6^th^ graders.

In Colorado, Healthy Kids was designed as an open communication format and followed a traditional motivational interviewing approach. Upon review of the program materials, the Alabama school teachers and researchers were concerned at the level of general reading literacy needed to comprehend the key messages. Specifically, terms such as bullying, depression, meditation, mindfulness, and self-care had different meanings and stigmatization in Alabama versus Colorado student communities. Due to these cultural differences, the content of the program materials was changed at the student level. The impact of this change was improved engagement from both teachers and school staff as the modified language was found to be more in line with their school culture.

Personnel modifications were also made to train local community members as program ALLYs. By utilizing an already-existing partnership between the school district and the local University, the aim was to increase the overall sustainability of the program. A total of eight ALLYs were trained *via* a virtual platform over the course of five, two-hour sessions (10 h training). The training included an introduction to the program and review of the pilot results; description of the ALLY’s role including relationship and communication skills with a cultural humility focus (see description of Empower Action Model below); and general background on motivational interviewing, reflecting and summarizing a conversation to provide focus, changing mindset and behaviors, and delivering a program under the umbrella of ethical and effective research (e.g., consent, documentation of good research practices, implementation science, intervention fidelity, etc.).

To ensure that ALLYs delivered a consistent intervention, the ALLYs and researchers partnered to create a program workbook. The new program format was comprised of specific weekly topics that would be covered during the one-to-one, 30-min sessions between the student and the ALLY facilitators. This modification resulted in more focused sessions that were easier for the ALLYs to teach with little prior knowledge on the program materials. The ALLYs reported feeling more confident in their ability to deliver the intervention compared to the practice sessions using the previous materials developed for the pilot program.

### Content modifications

Three content modifications were made: (1) adjustment of reading level, (2) adjustment of length of the intervention period, and (3) integration of a new framework that represented our more diverse community. Upon review of our program materials, the teachers and school counselors shared a concern that the reading level was too high for their 5^th^ and 6^th^ grade students. The average reading level of the students was reported to be at a 3^rd^ grade reading level. Thus, to ensure that students could understand and utilize our materials, we modified them from a 6th grade reading level to a 3rd grade level. Program facilitators found that students were able to read, comprehend, and complete the modified materials during the individual sessions.

According to data from our Colorado pilot, the necessary number of program sessions completed to elicit the most improvement of program outcomes was five or more sessions. The program was originally designed to be delivered in six, 30-min sessions which occurred over a six-week time period. In partnership with school staff and researchers, a modification was made to extend the length of the intervention period from 6 weeks to up to 15 weeks, thus spanning the full semester. This modification resulted in more students completing the full program, i.e., six sessions.

It was recognized that the majority of ALLYs did not represent the racial and ethnic background of the students (i.e., 12% of ALLYs identified as Black/African American). As a result, the research team along with the collaborators at Auburn University made a conscious effort to incorporate more cultural competency exercises and personal, self-assessments (i.e., cultural humility) including implicit bias recognition throughout the ALLYs training. Our cultural humility training was designed to prepare each ALLY for working within cross-cultural relationships including self-awareness, openness, and transcendence. This was taught using the ORCA-Stance framework including openness, respect, curiosity, and accountability. Each ALLY was required to complete the training and attend a group discussion for further learning and sharing of information. To guide the new training, a new framework was integrated into our program, the Empower Action Model (EAM) (37). The EAM extends the Socio-Ecological Model by targeting protective factors in a culturally-tailored delivery to reduce inequities among key program outcomes, including depression and anxiety (i.e., negative affectivity). ALLY was therefore modified to target improvements in all six Empower Action Model protective factor constructs: (1) build resilience, (2) create positive self-views, (3) grow through individual development, (4) share resources to meet basic needs, (5) support the formation of positive relationships, and, in doing such, (6) honor all cultural identities. These constructs were also included as key topics in our program materials for students to learn and development their skills.

To improve health equity of our program, we created a curriculum that focused on fostering social connectedness and self-identity of each study using the EAM model. More opportunities were incorporated for students to feel connected in their communities both as they see themselves, their family, and their community. We were shown through this work, that to create this environment, curriculum needs to be focused on improving individual-level protective factors (e.g., resilience, self-efficacy). By conducting individual sessions, ALLYs were able to empower each student to document supportive adult-child relationships, gain their own sense of perceived control within their lives, strengthen coping skills, and mobilize resources in their communities which were most personally meaningful to them (i.e., faith, gender, culture). We created new activities focused on improving students’ social connectedness, self-view, individual growth and development, awareness regarding available school and community resources to meet their basic needs, and knowledge on how to form supportive relationships in their family structure, school, and wider community.

## Discussion

The mental health of our nation’s youth has been slowly declining for years as suicidal behaviors and ideations continue to rise (38). The social isolation and lack of support services for our high need communities due to COVID-19 have made the situation an immediate public health crisis (24). The inequities in access to mental health services and overall mental health disparities continue to increase, and the rising levels of youth suicide among racially and ethnically minoritized youth is now a dire call for immediate action (39). School-based programs focused on reducing stress and symptoms of depression and anxiety have been effective; however, existing programs are hard to implement in the communities that may need the most help due to a lack of access or funding to deliver such programs (13, 40).

ALLY was created to combat these issues with little to no need for school resources by leveraging existing academic-community partnerships. This paper documents the various adaptations that our program implemented when it transitioned to a different state (Colorado to Alabama), community (urban to rural), and demographic composition (predominantly White, predominantly middle-class to predominantly Black, predominantly low-income). Although the format, one-to-one sessions for 30 min each week remained unchanged, several changes were noted. Major changes included: (1) the use of trusted, local community volunteers (ALLYs) to deliver program; (2) the challenge of varying levels of general and health-related literacy; and 3) the need to ensure programs are developed with program frameworks that focus on using an equity lens.

As previously documented, developing sustainable mental health programs in schools very much relies on a strong academic-community partnership (40). We chose to recruit our volunteers from Auburn University as local school districts had previously established partnerships with Auburn through which physical activity programs had been successfully delivered. The utilization of local community members without formalized mental health training to deliver community level interventions is not novel (19–21, 41–44). Programs focused on improving health behaviors (i.e., diet, physical activity) have been adequately delivered and sustained in communities using such approaches, with the added benefit of having people deliver the program in their own communities that have similar shared, life experiences as those receiving the program. Unfortunately, our volunteers did not represent the same racial or income group of our students. Moving forward we will focus efforts on having a more representative group deliver the program that aligns with the community demographics. Despite this limitation, our findings add to this existing literature by providing preliminary evidence that upstream, preventative mental health programs may also be feasibly delivered *via* ALLYs after a short training. To eliminate the barrier of limited available time among ALLYs due to busy schedules, we chose to deliver the training on a virtual platform with both synchronous and asynchronous sessions. Due to this, we were able to elicit participation from a larger group of volunteers with varying schedules because they could complete the majority of the training at a time that was most convenient to them. As COVID-19 has streamlined and normalized teaching and meetings in the virtual world, our training can easily be accessed beyond Colorado and Alabama. We believe that this training model has improved our ability to train new ALLYs efficiently and effectively and will facilitate program expansion and reach anywhere in the U.S. and even beyond.

The need to create program materials at appropriate levels for participants with a range of both general and health literacy is critical to the success of programs in high-needs communities (40). Our initial approach to combat this barrier was to create a student workbook with colorful activity sheets picturing many of the concepts and to modify all materials to be read at a 3rd grade reading level. This change was made during our training stage when even ALLYs, i.e., University students, expressed difficulty explaining and teaching program concepts during the practice sessions. The ALLYs reported that many of the concepts they were asked to teach students were also new to them and that more structure and examples were needed. The workbook was then created, and the practice sessions were redesigned utilizing the new materials. The ALLYs immediately reported increased confidence in their ability to deliver the program and knowledge of the educational activities. Further, the ALLYs reported using the program concepts in their own lives, thus suggesting that future research could examine the effect of the program on ALLYs mental health, as well, since the program benefits may extend beyond even the student participants.

In summary, ALLY is a universal, school-based, primary prevention intervention to promote mental well-being and to strengthen protective factors which has been adapted to meet the needs of a Title I elementary school in rural Alabama serving over 80% Black and Hispanic students. In this paper, we document the adaptations needed to deliver an existing program from an urban, middle-class, predominantly White community in Colorado to a rural, low-income, racially and ethnically diverse community in Alabama. A separate manuscript we will document pre-post changes in key mental health indicators to ensure that we can attain similar or superior program outcomes as in our Colorado pilot. Future work also needs to consider the level of supervision and ongoing training required to maintain effective ALLYs, including continued didactic training, case discussions, role-playing, as well as adherence ratings to the program using live or video observational fidelity measures (18).

## Data availability statement

The original contributions presented in the study are included in the article/supplementary materials, further inquiries can be directed to the corresponding author.

## Ethics statement

The studies involving human participants were reviewed and approved by Colorado Institutional Review Board. Written informed consent from the participants’ legal guardian/next of kin was not required to participate in this study in accordance with the national legislation and the institutional requirements.

## Author contributions

JK wrote the first draft of the manuscript. AB, SS, AD, JC, RM, and MP contributed and approved the final manuscript. All authors agreed to be accountable for all aspects of the work.

## Conflict of interest

The authors declare that the research was conducted in the absence of any commercial or financial relationships that could be construed as a potential conflict of interest.

## Publisher’s note

All claims expressed in this article are solely those of the authors and do not necessarily represent those of their affiliated organizations, or those of the publisher, the editors and the reviewers. Any product that may be evaluated in this article, or claim that may be made by its manufacturer, is not guaranteed or endorsed by the publisher.
